# Immune indices and oral health in patients infected with the human immunodeficiency virus

**DOI:** 10.1186/s12903-023-03752-y

**Published:** 2023-12-15

**Authors:** Yuxiang Yang, Feixue Yu, Yujie Fei, Guangyan Dong, Peilin Cao, Yi Liu

**Affiliations:** 1 Department of Radiology, West China School of Public Health and West China Fourth Hospital, Sichuan University, Chengdu, 610041 Sichuan China; 2 West China School of Stomatology, Sichuan University, Chengdu, 610041 Sichuan China; 3 State Key Laboratory of Oral Diseases, West China Hospital of Stomatology, National Clinical Research Center for Oral Diseases, Sichuan University, Chengdu, 610041 Sichuan China; 4 Department of Stomatology, Sichuan Provincial People’s Hospital, University of Electronic Science and Technology of China, 32# W. Sec 2, 1st Ring Rd. Chengdu, Chengdu, 610072 Sichuan China

**Keywords:** Human immunodeficiency virus, Demography, Dental health, CD4^+^ T-cell count, Human immunodeficiency virus viral load

## Abstract

**Background:**

The human immunodeficiency virus (HIV) is the causative agent of acquired immunodeficiency syndrome (AIDS). During the incubation period of AIDS, oral manifestations may precede systemic symptoms; therefore, it is vitally important to explore the relationship between HIV and oral health and other indicators. This study aimed to further assess the correlation between demographic risk factors, the dental health of HIV-infected patients, and the correlation of oral health indicators with CD4^+^ T-cell counts (CTCCs) and HIV viral loads (HIV-VLs).

**Methods:**

Demographic data on 108 HIV-infected patients were first recorded by questionnaire from March 2016 to November 2018. Patients’ dental health and oral lesions were assessed by a dental specialist; in addition, they were tested for CTCCs and HIV-VLs by flow cytometry and NucliSENS EasyQ® HIV-1 virometer. Finally, the links between CTCC, HIV-VL, and the dental health (including oral lesions) of the patients were analyzed.

**Results:**

We found that age, marital status, and body mass index (BMI) were relevant to the patient’s dental health (*P* < 0.05) and that their oral hygiene was relevant to their dental health (*P* < 0.05). However, HIV-VL was not directly related to periodontal/dental clinical indicators (*P* > 0.05). We discovered that the oral lesions in HIV-infected patients were related to decreased CTCCs and increased HIV-VLs (*P* < 0.05).

**Conclusions:**

We concluded that HIV-infected patients with severely impaired immune function tend to have poor dental health. Moreover, the prevalence of oral lesions was negatively correlated with CTCC and positively correlated with HIV-VL.

**Supplementary Information:**

The online version contains supplementary material available at 10.1186/s12903-023-03752-y.

## Background

Acquired immune deficiency syndrome (AIDS) is a characteristic immune deficiency disease caused by infection with the human immunodeficiency virus (HIV); it is a highly infectious disease that spreads rapidly and has a high mortality rate [1]. HIV is a retrovirus that targets CD4^+^ T cells in the immune system, leading to impaired immune cell function and severe immunosuppression [2]. Although there is no cure for AIDS, there are palliative drugs that can enable patients to live with HIV [3]. Microorganisms in the oral cavity of AIDS patients are diverse and complex [4, 5]. Compared with non-HIV-infected patients, patients with HIV have a higher risk of developing AIDS and worsening oral disease [6]. However, the exact mechanisms linking oral disease with AIDS are not fully understood.

CD4^+^ T cells, which are mainly expressed in helper T (Th) cells, are donor receptors for Th cells, enabling them to recognize antigens from the T-cell receptor (TCR) [7]. CD4^+^ T cells bind to the nonpolypeptide region of the major histocompatibility complex (MHC) and participate in the signal transduction of TCR-recognized antigens in Th cells [8]. CD4^+^ T cells are also receptors for HIV [9]. Therefore, the detection of CD4^+^ T cells plays a crucial role in confirming the effectiveness of both AIDS therapy and patients’ immune function. Currently, the CD4^+^ T-cell count (CTCC) is also the clearest indicator of immune system damage in HIV-infected patients [10].

The HIV-VL assay is applied mainly to monitor the progression of HIV infection [11]. At present, the most commonly used and most sensitive method is the quantitative detection of viral RNA in plasma. Therefore, further comparison between CTCCs and HIV-VLs with oral health indicators is essential for the control of disease in AIDS patients.

## Methods

### Patient information

Our study comprised 108 patients (average age, 39.8 years; age range, 13–86 years) with either HIV infection or full-blown AIDS who had been diagnosed in the HIV department of the Public Health Clinical Medical Center of Chengdu, Sichuan Province, from March 2016 to November 2018. The inclusion criteria were as follows: patients had to be at least 13 years of age; they had to be able to think, to have normal speech, to be able to express themselves, and to meet the diagnostic criteria for HIV infection (i.e., a positive HIV antibody test with secondary confirmation by rapid or laboratory immune-enzymatic techniques and/or a positive test for HIV or its viral components [HIV-RNA or HIV-DNA or ultrasensitive HIV p24 antigen]). The exclusion criteria included pregnancy in women, the presence of a severe opportunistic infection uncontrollable by treatment, the individual’s inability to move independently, and any other serious or unstable chronic medical condition requiring care. All included patients gave their informed consent and participated voluntarily; in the case of minors, their parents/legal guardians also signed the consent form. All subjects filled out an epidemiologic questionnaire before undergoing a clinical examination. This study was performed by the Declaration of Helsinki and also received the approval of the ethics committee at West China Hospital of Stomatology, Sichuan University (WCHSIRB-D-2015-004).

### Demographics

The participants’ demographics were obtained by questionnaire, and their general health was determined from their inpatient medical records. Based on the criteria of the World Health Organization (WHO), the assessed variables included gender, age, education, income, and body mass index (BMI). The study population was split into four groups: adolescents (13–24 years of age), a young group (25–44 years of age), a middle-aged group (45–64 years of age), and an elderly group (65 years of age or older). The level of education was classified as follows: junior middle school (< 9 years), high school (9–12 years), or college or beyond (> 12 years). Annual income—in terms of renminbi (RMB)-was split into less than 12,000, 12,000 to 36,000, and > 36,000 RMB. Marital status was classified as single, married, divorced, or widowed. Based on their BMI (weight [kg]/height [m]^2^), participants were classified as either underweight (< 18.5 kg/m^2^), normal (18.5–23.9 kg/m^2^), overweight (24.0–27.9 kg/m^2^), or obese (≥ 28.0 kg/m^2^) [12].

### Clinical assessment of dental health

Each of the patients received a comprehensive dental examination. We applied the WHO’s Basic Methods for Oral Health Survey as well as the basic methods and diagnostic criteria of the Second National Oral Health Epidemiological Sample Survey [13]. In each case, the dental examination determined the number of sound permanent teeth; the number of teeth missing owing to oral disease; the number of decayed, missing, and filled teeth (DMFTs); and several decayed, missing, and filled surfaces (DMFSs). The dental health examination was performed by an oral surgeon (who had undergone prior training) with a consistency test and kappa value greater than 0.8. Sterilization of the examination instruments and personnel protection measures were carried out based on the requirements of the HIV department. Twenty-seven patients were examined each time, and the HIV department disposed of medical waste.

### Clinical assessment of periodontal health

All of the patients underwent a comprehensive periodontal examination using a Hu-Friedy periodontal probe and disposable mouth mirror. The examination was performed by an oral surgeon based on Carranza’s Clinical Periodontology method [14]. The examination indexes included probing depth (PD), clinical attachment loss (CAL [> 3 mm and > 5 mm]), PD > 4 mm, and bleeding on probing (BOP). For PD and CAL, six loci (proximal mid-buccal, buccal, distal midbuccal, distal midlingual, lingual, and proximal midlingual) of each tooth were evaluated using a William probe (Hu-Friedy, Chicago, USA). For grading BOP, four loci (distal midbuccal, medial, proximal midbuccal, and lingual) were examined, with 0 = no bleeding and 1 = bleeding.

### Measurement of CD4^+^ T-cell count and HIV viral loads

Ten milliliters of peripheral venous blood (anticoagulated with ethylenediaminetetraacetic acid) were collected by a specialist nurse using a sterile blood collection tube; the specimen was then processed within 24 h and sent to the laboratory for testing. The CD4^+^ T-cell count (CTCC) in peripheral blood was measured by flow cytometry (BD Biosciences, San José, CA, USA). After extracting HIV RNA from plasma, the HIV viral load was detected by using the NucliSENS magnetic extraction reagent and HIV type I nucleic acid quantitative kit on the NucliSENS EasyQ HIV-1 virometer (bioMerieux, Marcy-l’Etoile, France). Subsequently, patients were classified in terms of viral load (i.e., < 10,000 copies/mL [low-VL group], 10,000 − 100,000 copies/mL [median VL group], and ≥ 100,000 copies/mL [high-VL group]). In addition, the patients were split into high- and low-CTCC groups (200/µL).

### Diagnostic criteria for oral lesions

The diagnosis of oral lesions was based on the classification and diagnostic criteria of AIDS oral manifestations formulated by the WHO Collaborating Centre on Oral Manifestations of the Immunodeficiency Virus [15]. HIV-associated oral lesions primarily include oral candidiasis (OC), oral hairy leukoplakia (OHL), herpes simplex (HS) lesions, Kaposi’s sarcoma (KS), non-Hodgkin’s Kaposi’s sarcoma (KS), non-Hodgkin’s lymphoma (NHL), periodontal diseases (linear erythema of the gums, necrotizing ulcerative gingivitis, and necrotizing ulcerative periodontitis), xerostomia, and others [16]. The diagnosis of oral lesions was based on clinical presentation and histopathologic examination. The examination of oral lesions was completed by an oral surgeon according to unified examination methods and standards. For the diagnosis of non-Hodgkin’s lymphoma, the histological sections were stained with hematoxylin and eosin (H&E), which were confirmed by at least two oral pathologists based on the WHO classification of lymphoid neoplasms [17].

### Statistical analysis

The data were analyzed using SPSS 23.0 software (SPSS, Inc., Chicago, IL, USA). Comparisons of multiple samples were performed using the nonparametric Kruskal–Wallis test. Comparisons of two samples were performed using independent samples and the Mann–Whitney U test. *P* < 0.05 denoted a significant difference.

## Results

### Treatment-naive and experienced HIV-infected patients

Our 108 patients were divided into those who were treatment-naive (*n* = 38) and those who had experienced treatment (*n* = 70). Because we found that there was no significant difference in the number of permanent sound teeth, missing teeth from disease, DMFTs, and DMFSs between untreated and treated groups (Figure S1), we decided to refer to them all simply as HIV-infected patients.

### Dental health status of HIV-infected patients

Among the caries-related indicators, the mean number of existing healthy teeth was 10.8, with individual fluctuations ranging from 0 to 23, and the mean number of teeth lost due to oral disease was 5.6, with individual fluctuations ranging from 0 to 21. The mean DMFT value was 11.8, with individual fluctuations ranging from 1 to 26, and the mean DMFS value was 47.6, with individual fluctuations ranging from 2 to 112 (Table 1).

**Table 1:** Clinical index of dental health in HIV-infected patients (N = 108)

Clinical parameters	Min	Max	Mean ± Std Dev
Number of permanent sound teeth	0	23	10.8 ± 6.3
Number of missing teeth from disease	0	21	5.7 ± 2.2
DMFT	1	26	11.8 ± 6.3
DMFS	2	112	47.6 ± 28.3

### Statistical analysis of demographic variables and dental clinical indicators in HIV-infected patients

We also analyzed the relationship between four clinical indicators of dental health and demographic variables. The results showed that, owing to oral diseases and the higher number of DMFSs, the older group had fewer remaining teeth than the younger group (*P* < 0.05). Significant differences were also found in the number of existing teeth, DMFTs, and DMFSs in terms of marital status (*P* < 0.05). Both divorced and widowed individuals had higher DMFTs and DMFSs than did single and married individuals. Gender, education, and income were not significantly correlated with the previously mentioned clinical indicators. The number of teeth lost due to oral disease and DMFTs differed significantly between different BMI groups (*P* < 0.05) (Table 2).

**Table 2:** Comparison of demographic variables and dental health status in HIV-infected patients (N = 108)

Variables	N	Mean number of permanent sound teeth	Mean number of missing teeth from disease	DMFT	DMFS
				mean	mean
Age (yrs)	108				
13 ~ 24	26	14.17	4.37	10.37	42.43
25 ~ 44	32	14.12	4.24	11.18	44.63
45 ~ 64	29	11.62	6.79	13.82	52.71
≥ 65	21	9.89	8.51	15.09	62.91
	*P*	0.034^**^	0.004^**^	0.036^**^	0.013^**^
Gender	108				
Male	50	13.79	5.09	11.78	45.55
Female	58	11.85	6.22	13.24	53.28
	*P*	0.225	0.932	0.361	0.338
Marital status	108				
Single	19	2.23	1.5	8.42	19.16
Married	45	3.12	2.9	14.23	31.59
Divorced	25	3.68	3.47	18.63	35.02
Widowed	19	3.24	2.68	20.31	31.13
	*P*	0.004^**^	0.001^**^	0.038^*^	0.088
Education (yrs)	108				
< 9	43	12.19	6.61	13.95	46.06
9 ~ 12	46	13.42	5.44	11.67	50.69
> 12	19	12.16	5.43	12.48	55.36
	*P*	0.735	0.898	0.561	0.466
Income per year (yuan)	108				
< 12,000	12	12.74	6.57	13.15	19.93
> 12,000	96	11.87	5.47	12.14	53.52
	*P*	0.654	0.445	0.796	0.645
BMI (kg/m2)	108				
< 18.5	25	14.48	3.42	11.24	41.02
18.5 ~ 24	78	11.21	4.98	14.27	49.42
24 ~ 28	5	15.49	10.15	16.01	62.46
	*P*	0.123	0.007**	0.037**	0.056

*P < 0.05; **P < 0.01

### Influence of oral hygiene on patients’ dental health

We discovered no significant association between the frequency of tooth brushing or flossing frequency and the clinical indicators of caries; mouth rinsing frequency was significantly associated with the number of permanent sound teeth and DMFTs (*P* < 0.05). Those who used mouthwash more than once a day had the most permanent sound teeth and the fewest DMFTs (Table 3).

**Table 3:** Effect of oral hygiene behaviors on dental health status in HIV-infected patients (N = 108)

Variables	N	Mean number of permanent sound teeth	Mean number of missing teeth from disease	DMFT mean	DMFS mean
Frequency of tooth brushing	108				
More than once a day		12.07	6.05	13.21	52.71
Once a day		16.19	4.62	9.24	36.18
	*P*	0.073	0.621	0.056	0.114
Flossing frequency					
More than once a day		11.52	4.58	12.77	54.01
Once a day		11.25	5.34	13.47	58.42
Every few days		13.25	5.42	12.23	48.26
Seldom/Never		13.46	6.76	12.43	45.62
	*P*	0.673	0.581	0.908	0.376
Mouth rinsing frequency					
Seldom/Never		9.26	8.91	16.32	62.01
Every few days		13.62	5.35	11.38	52.95
Once a day		11.51	3.22	12.31	51.01
More than once a day		14.56	4.61	10.95	42.83
	*P*	0.039*	0.064	0.018*	0.107

*P < 0.05

### Patients’ CD4^+^ T-cell counts and HIV viral loads

The minimal CTCC in the patients was 2 cells/µl and the maximal value was 733 cells/µl. The minimal HIV-VL in the patients was 167 copies per milliliter and the maximal HIV-VL was 9,580,000 copies per milliliter (Table 4). Both the HIV-VL and CTCC were further counted in untreated and treated groups using T-test analysis; the resulting data showed that the HIV-VL (copies per milliliter) was notably decreased in the treated group as compared with the untreated group (Figure S2A). Furthermore, the CTCC (cells/µl) was dramatically increased in the treated group as compared with the untreated group (Figure S2B).

**Table 4:** CTCC and HIV-VL in HIV-infected patients (N = 108)

Parameters	Values
HIV-VL (copies/ml)	
VL < 50	0 (0%)
167 < VL ≤ 10,000	30 (27.8%)
10,000 < VL ≤ 100,000	16 (14.8%)
VL > 100,000	62 (57.4%)
CTCC (cell/µl)	
CTCC ≥ 350	12 (11.1%)
200 < CTCC < 350	10 (9.3%)
50 < CTCC < 200	33 (30.6%)
CTCC < 50	53 (49.1%)

### The effects of the CD4^+^ T-cell count and HIV viral load on patients’ periodontal health

The patients were grouped based on their CTCCs and HIV-VLs; the differences in their periodontal health indices were then compared, showing that there was no statistical association between any of the periodontal health indices and patients’ HIV-VLs (*P* > 0.05). However, there were statistical associations between patients’ CTCCs and their percentage of bleeding on probing (BOP) positive sites, probing depth (PD > 4 mm), mean PD, and clinical attachment loss (CAL > 3 mm and CAL > 5 mm) (Table 5).

**Table 5:** Comparison of HIV-VL and CTCC on periodontal health indicators in HIV-infected patients (N = 108)

Parameters	N	BOP (%)	Mean Probing Depth (mm)	Mean CAL (mm)	PD > 4 mm sites (%)	CAL > 3 mm sites (%)	CAL > 5 mm sites (%)
HIV-VL (copies/ml)							
167 ~ 10,000	30	34.65	2.68	2.39	13.19	26.02	7.93
10,000 ~ 100,000	16	33.72	3.19	3.12	22.46	33.97	11.19
> 100,000	62	33.12	3.22	2.89	14.74	30.89	10.11
	*P*	0.832	0.382	0.372	0.285	0.371	0.393
CTCC (cells/µl)							
> 350	12	47.57	2.49	2.47	6.82	22.23	5.19
≤ 350	96	29.79	3.26	2.91	17.35	32.19	10.78
	*P*	0.000**	0.047*	0.269	0.002**	0.014*	0.002**

*P < 0.05; **P < 0.01

### The effects of the CD4^+^ T-cell count and HIV viral load on patients’ dental health

We further compared the differences between CTCCs and HIV-VLs and the dental health of the patients. The resulting data showed that the following *P* values—0.981, 0.540, 0.089, and 0.952 (> 0.05)-corresponded with permanent sound teeth, missing teeth from disease, DMFTs, and DMFSs, respectively. Therefore, there was no significant difference between the four indicators in the different HIV-VLs. Similarly, there was no significant difference between the CTCCs and the indicators of permanent sound teeth and missing teeth from disease. The *P* values for DMFTs and DMFSs in the CTCC grouping were 0.013 and 0.014, respectively, indicating significant differences between DMFTs or DMFSs and CTCCs (Table 6).

**Table 6:** Comparison of HIV-VL and CTCC on dental health indicators in HIV-infected patients (N = 108)

Parameters	N	Mean number of permanent sound teeth	Mean number of missing teeth from disease	DMFT mean	DMFS mean
HIV-VL (copies/ml)		9.528	5.269	12.019	12.454
167 ~ 10,000	30	9.533	5.567	12.500	12.333
10,000 ~ 100,000	16	9.750	5.688	12.938	12.500
> 100,000	62	9.468	5.016	11.548	12.500
	*P*	0.981	0.540	0.089	0.952
CTCC (cells/µl)		9.528	5.269	12.019	12.454
> 350	12	8.750	5.750	13.833	10.750
≤ 350	96	9.625	5.208	11.792	12.667
	*P*	0.485	0.485	0.013*	0.014*

*P < 0.05

### The effects of the CD4^+^ T-cell count and HIV viral load on patients’ oral lesions

Finally, we further compared CTCCs and HIV-VL with the indicators related to oral lesions, which have been identified in our previous study [18]. By using the chi-square test, we found that the *P* values of oral lesions in the HIV-VL and CTCC subgroups were 0.009, 0.709, and 0.019, respectively, or < 0.05, indicating that there was a significant difference between the HIV-VL or CTCC and oral lesions. These results show that the incidence of oral lesions was significantly higher in the CTCC group with more than 200 cells/µl than in the group with less than that number. Additionally, an HIV-VL of more than 100,000 copies per milliliter was associated with the presence of oral lesions, whereas an HIV-VL between 167 and 10,000 copies per milliliter was associated with the absence of oral lesions (Table 7).

**Table 7:** Influence of CTCC and HIV-VL on oral lesions in HIV-infected patients (N = 108)

Parameters	HIV-associated oral lesions	No HIV-associated oral lesions	X^2^ value	*P*
CTCC (cells/µl)				
< 200	44	40	5.510	0.019*
≥ 200	19	5		
HIV-VL (copies/ml)				
167 ~ 10,000	12	18	9.476	0.009**
10,000 ~ 100,000	10	11		
> 100,000	41	16		

*P < 0.05; **P < 0.01

## Discussion

To assess their oral health status and immune indices and to collect the patient’s demographic characteristics, we adopted a questionnaire. All patients were first classified as either treatment-naive or experienced, with the data indicating no difference in oral health indicators (permanent sound teeth, missing teeth from disease, DMFTs, and DMFSs) between treatment-naive and experienced patients. Thus, both groups were treated alike in the subsequent analysis, which showed that, due to oral diseases and DMFSs, older patients had fewer remaining teeth. This suggested that with advancing age, dental health declines due to oral diseases. Both DMFTs and DMFSs were higher among the divorced and widowed individuals than among the single or married. This indicated that being divorced or widowed was associated with poor dental health, which may be related to mental stress due to such factors as family breakdown and economic pressure. Moreover, the data revealed that a higher BMI was associated with more missing teeth from disease and DMFTs. This may stem from the fact that people in different BMI groups have different dietary habits and that a high BMI is related to the excessive consumption of sweets and sugary foods associated with the development of caries. Additionally, the data showed that the frequent use of mouthwash was significantly associated with a high number of existing healthy teeth and a low number of DMFTs. Gender, educational level, income level, tooth brushing frequency, flossing frequency, smoking, and alcohol consumption were not significantly associated with patients’ dental health status. Oral hygiene through regular tooth brushing is the mainstay of controlling plaque deposits contributing to tooth decay (caries). Normally, tooth brushing frequency and flossing frequency should be correlated with caries. The reason for this contradiction may be that we are studying specific patients (HIV patients); the number of cases in the current study is not large enough; and individual differences in patients.

Periodontal disease is a mixed microbial disease affecting the periodontal supporting tissues [19]. Its pathologic characteristics are reflected mainly in the interaction between certain anaerobic bacteria and the host immune system, eventually leading to the degradation of the periodontal tissues [20]. Studies have shown that there is a correlation between periodontal disease and HIV infection [21, 22]. Furthermore, the onset and progression of periodontal disease have been reported to be associated with the degradation of the immune system [23]. As the data from our previous study showed, age, marital status, education, and annual income were associated with several clinical indicators of periodontal; sex was associated only with probing depth (PD) and clinical attachment loss (CAL); rural-dwelling patients were associated with high CAL; and high BMI was associated with high bleeding on probing (BOP) and PD [18]. It was reported that the normal level of CTCCs in adults is 844 ± 247 cells per cubic millimeter [24]; however, this commonly fluctuates widely with physiologic conditions, and the ratio of CD4^+^ to CD8^+^ cells in our study was 1.2 to 1.9:1 [25]. The frequency of opportunistic infections in the patients was related to their CTCC and CD4/CD8 ratios. A CTCC level of less than 200 cells/µl and a CD4/CD8 ratio below 0.20 were associated with a significant increase in HIV opportunistic infections [26]. The CTCC level can reflect an individual’s immune status and the progression of the disease; it can also help clinicians evaluate therapeutic effects [27]. The occurrence and development of periodontal diseases are closely related to the function of the immune system [28, 29]. Our study confirmed that there were statistically significant differences in the periodontal health indicators between CTCC > 200 cells/µl and ≤ 350 cells/µl groups. The BOP of the CTCC > 350 cells/µl group was lower than those of the CTCC ≤ 200 cells/µl group; whereas their mean PD values (PD > 4 mm, CAL > 3 mm, and CAL > 5 mm) were higher than those of CTCC > 350 cells/µl. These data suggest that the lower the CTCC, the heavier the clinical indicators associated with periodontitis, while the gingival bleeding symptoms of gingivitis are instead reduced. Vastardis et al. found that when CTCC > 500 cells/µl, there was no statistically significant association between CTCC and clinical periodontal index, and gingival inflammation was lower than expected [30]. Another study also found no significant association between periodontal variables and the CTCC [31]. Advances in therapeutic strategies and an emphasis on oral hygiene may be responsible for this change in the incidence of associated lesions. A recent study also suggests that HIV infection may increase the risk of chronic periodontitis [32]. The vast majority of AIDS patients involved in this part of the study had severe disease and their CTCCs were much lower than normal, posing a high risk of severe periodontal destruction. In our results, a CAL > 3 mm and CAL > 5 mm, reflecting the severity of periodontitis, were higher in the group with low levels of CD4^+^ T cells than in the group with higher levels. Most scholars believe that HIV-infected individuals are at increased risk for caries. Caries susceptibility increases with increasing HIV symptoms and immunosuppression and decreasing CD4 + T cells, and most HIV-infected individuals are prone to violent caries [28, 29].

Our study also found that the HIV-VL was not correlated with clinical indicators of periodontal health [33]. The HIV-VL in plasma can objectively and directly reflect the state of viral replication in HIV-infected patients. Based on a study of the viral load [34], we classified 108 patients into the following groups: 167 to 10,000 copies per microliter (low); 10,000 to 100,000 copies per microliter (medium), and more than 100,000 copies per microliter (high). The number of cases in the “high” group accounted for more than half (57.4%) of the total, and the process of viral replication in this group was highly active. Our data further showed that all dental clinical indicators were unassociated with HIV-VL. We also demonstrated an association between the incidence of oral opportunistic infections characteristic of HIV infection and the HIV-VL or CTCC [10].

During HIV infection, most patients often develop oral lesions, which may be the first symptom [21]. Study revealed that the occurrence rate of oral lesions in AIDS patients is 2-2.5 times higher than that in the general population, and 70–90% of HIV patients have at least one oral representation [35]. Oral representation is a prominent feature of HIV infection and AIDS [36, 37]. Our previous study also revealed that the oral manifestations of these 108 HIV-infected patients were predominantly characterized by candidiasis albicans, salivary gland disease, AIDS-related periodontitis, and oral ulcers; fewer had oral hairy leukoplakia, herpes simplex stomatitis phenotypes, and there were no patients who suffered from lymphadenopathy, Kaposi’s sarcoma, or non-Hodgkin’s lymphoma [18]. The current study further proved that HIV-associated oral lesions were higher in the CTCC (< 200 cells/µl) group than in the CTCC (≥ 200 cells/µl) group. The results of this study are consistent with previous findings that the prevalence of oral lesions in HIV-infected patients is negatively correlated with CTCC and positively correlated with HIV-VL [38]. Thus, HIV-VL and CTCC can be used as predictors of oral lesions in HIV-infected patients.

Therefore, we conducted a detailed, comprehensive assessment of the patient’s oral health to elucidate the association between oral health and HIV infection. The resulting data indicate that the oral health of HIV-infected patients is relatively poor and that most of them have oral lesions. Age, BMI index, and CTCC were associated with patients’ oral health. Therefore, clinicians should pay attention to oral health in the diagnosis and treatment of HIV-infected patients to reduce the prevalence of pathogens and thus better serve their patients.

It should be noted that the sensitivity and specificity of our results were not very high, which was related to our small sample size. Besides, the collection of clinical data was not sufficient. Therefore, increasing the sample size and observational items will greatly improve the accuracy of the study, which will be more useful for clinical guidance.

## Conclusions

Our data show that HIV-infected patients with severe immune deficiencies have relatively poor periodontal and dental health and are more likely than others to develop oral diseases. By comparing the association of CTCCs and HIV-VLs with oral health indicators in HIV-infected patients, we concluded that these values could serve as indexes to predict the effects of dental treatment.

### Electronic supplementary material

Below is the link to the electronic supplementary material.

Supplementary Material 1

Supplementary Material 2

Supplementary Material 3

## Data Availability

Data and research materials from this study will be available to the corresponding author upon reasonable request.

## Abbreviations

HIV: Human immunodeficiency virus
CTCC: CD4^+^ T-cell count
HIV-VL: HIV viral load
BMI: Body mass index
AIDS: Acquired immune deficiency syndrome
Th: Helper T
WHO: World Health Organization
DMFT: Filled teeth
DMFS: Filled surfaces
PD: Probing Depth (PD)
CAL: Clinical attachment loss
BOP: Bleeding on probing

## References

[1] Whiteside A, Wilson D (2018). Health and AIDS in 2019 and beyond. Afr J AIDS Res.

[2] Ratnam M, Nayyar AS, Reddy DS, Ruparani B, Chalapathi K, Azmi SM (2018). CD4 cell counts and oral manifestations in HIV infected and AIDS patients. J Oral Maxillofacial Pathology: JOMFP.

[3] Cao W, Hsieh E, Li T (2020). Optimizing treatment for adults with HIV/AIDS in China: successes over two decades and remaining challenges. Curr HIV/AIDS Rep.

[4] Aškinytė D, Matulionytė R, Rimkevičius A (2015). Oral manifestations of HIV Disease: a review. Stomatologija.

[5] Lomelí-Martínez SM, González-Hernández LA, Ruiz-Anaya AdJ, Lomelí-Martínez MA, Martínez-Salazar SY, Mercado González AE (2022). Oral manifestations Associated with HIV/AIDS patients. Medicina.

[6] Weinberg A, Tugizov S, Pandiyan P, Jin G, Rakshit S, Vyakarnam A (2020). Innate immune mechanisms to oral pathogens in oral mucosa of HIV-infected individuals. Oral Dis.

[7] Schneidman-Duhovny D, Khuri N, Dong GQ, Winter MB, Shifrut E, Friedman N (2018). Predicting CD4 T-cell epitopes based on antigen cleavage, MHCII presentation, and TCR recognition. PLoS ONE.

[8] Saigusa R, Roy P, Freuchet A, Gulati R, Ghosheh Y, Armstrong Suthahar SS (2022). Single cell transcriptomics and TCR reconstruction reveal CD4 T cell response to MHC-II-restricted APOB epitope in human Cardiovascular Disease. Nat Cardiovasc Res.

[9] Neidleman J, Luo X, Frouard J, Xie G, Hsiao F, Ma T (2020). Phenotypic analysis of the unstimulated in vivo HIV CD4 T cell reservoir. Elife.

[10] Ottria L, Lauritano D, Oberti L, Candotto V, Cura F, Tagliabue A (2018). Prevalence of HIV-related oral manifestations and their association with HAART and CD4 + T cell count: a review. J Biol Regul Homeost Agents.

[11] Drain PK, Dorward J, Bender A, Lillis L, Marinucci F, Sacks J (2019). Point-of-care HIV viral load testing: an essential tool for a sustainable global HIV/AIDS response. Clin Microbiol Rev.

[12] Consultation WHOE (2004). Appropriate body-mass index for Asian populations and its implications for policy and intervention strategies. Lancet.

[13] Wang HY, Petersen PE, Bian JY, Zhang BX (2002). The second national survey of oral health status of children and adults in China. Int Dent J.

[14] Newman MG, Takei H, Klokkevold PR, Carranza FA. Carranza’s Clinical Periodontology. Elsevier Health Sciences; 2011.

[15] Classification. and diagnostic criteria for oral lesions in HIV infection. EC-Clearinghouse on Oral Problems Related to HIV Infection and WHO Collaborating Centre on Oral Manifestations of the Immunodeficiency Virus. J Oral Pathol Med. 1993;22(7):289 – 91. 8229864

[16] Patton LL, Phelan JA, Ramos-Gomez FJ, Nittayananta W, Shiboski CH, Mbuguye TL (2002). Prevalence and classification of HIV-associated oral lesions. Oral Dis.

[17] Swerdlow SH, Campo E, Pileri SA, Harris NL, Stein H, Siebert R (2016). The 2016 revision of the World Health Organization classification of lymphoid Neoplasms. Blood.

[18] Cao P, Zhang Y, Dong G, Wu H, Yang Y, Liu Y (2022). Clinical oral Condition Analysis and the influence of highly active antiretroviral therapy on human salivary Microbial Community Diversity in HIV-Infected/AIDS patients. Front Cell Infect Microbiol.

[19] Liccardo D, Cannavo A, Spagnuolo G, Ferrara N, Cittadini A, Rengo C et al. Periodontal Disease: a risk factor for Diabetes and Cardiovascular Disease. Int J Mol Sci. 2019;20(6). 10.3390/ijms20061414 PMC6470716 30897827

[20] Curtis MA, Diaz PI, Van Dyke TE (2020). The role of the microbiota in periodontal Disease. Periodontol 2000.

[21] Lomelí-Martínez SM, González-Hernández LA, Ruiz-Anaya AJ, Lomelí-Martínez MA, Martínez-Salazar SY, Mercado González AE et al. Oral manifestations Associated with HIV/AIDS patients. Med (Kaunas). 2022;58(9). 10.3390/medicina58091214 PMC9504409 36143891

[22] Valian NK, Houshmand B, Ardakani MT, Mahmoudi S. Microbiological Study of Periodontal Disease in populations with HIV: a systematic review and Meta-analysis. Clin Lab. 2023;69(5). 10.7754/Clin.Lab.2022.220738 37145081

[23] Becerra–Ruiz JS, Guerrero–Velázquez C, Martínez-Esquivias F, Martínez‐Pérez LA, Guzmán–Flores JM (2022). Innate and adaptive immunity of periodontal Disease. From etiology to alveolar bone loss. Oral Dis.

[24] Achhra AC, Zhou J, Dabis F, Pujari S, Thiebaut R, Law MG (2010). Difference in absolute CD4 + count according to CD4% between Asian and caucasian HIV-infected patients. J AIDS Clin Res.

[25] Castilho JL, Bian A, Jenkins CA, Shepherd BE, Sigel K, Gill MJ (2022). CD4/CD8 ratio and cancer risk among adults with HIV. JNCI: J Natl Cancer Inst.

[26] Mutoh Y, Nishijima T, Inaba Y, Tanaka N, Kikuchi Y, Gatanaga H (2018). Incomplete recovery of CD4 cell count, CD4%, and CD4/CD8 ratio in patients with human immunodeficiency virus Infection and suppressed viremia during long-term antiretroviral therapy. Clin Infect Dis.

[27] Figueredo CM, Lira-Junior R, Love RM. T and B cells in Periodontal Disease: New functions in a Complex scenario. Int J Mol Sci. 2019;20(16). 10.3390/ijms20163949 PMC6720661 31416146

[28] Mellors JW, Rinaldo CR, Gupta P, White RM, Todd JA, Kingsley LA (1996). Prognosis in HIV-1 Infection predicted by the quantity of virus in plasma. Science.

[29] Mellors JW, Munoz A, Giorgi JV, Margolick JB, Tassoni CJ, Gupta P (1997). Plasma viral load and CD4 + lymphocytes as prognostic markers of HIV-1 Infection. Ann Intern Med.

[30] Vastardis SA, Yukna RA, Fidel PL, Leigh JE, Mercante DE (2003). Periodontal Disease in HIV-positive individuals: association of periodontal indices with stages of HIV Disease. J Periodontol.

[31] Mulligan R, Phelan JA, Brunelle J, Redford M, Pogoda JM, Nelson E (2004). Baseline characteristics of participants in the oral health component of the women’s interagency HIV Study. Commun Dent Oral Epidemiol.

[32] Pólvora TLS, Nobre ÁVV, Tirapelli C, Taba M, Macedo LD, Santana RC (2018). Relationship between human immunodeficiency virus (HIV-1) Infection and chronic periodontitis. Expert Rev Clin Immunol.

[33] John C, Stephen L, Joyce Africa C (2013). Is human immunodeficiency virus (HIV) stage an Independent risk factor for altering the periodontal status of HIV-positive patients? A South African study. BMC Oral Health.

[34] Bucciardini R, Pugliese K, Weimer L, Digregorio M, Fragola V, Mancini M (2014). Relationship between health-related quality of life measures and high HIV viral load in HIV-infected triple-class-experienced patients. HIV Clin Trial.

[35] Sharma G, Pai KM, Setty S, Ramapuram JT, Nagpal A (2009). Oral manifestations as predictors of immune suppression in a HIV-/AIDS-infected population in south India. Clin Oral Investig.

[36] Gottlieb MS, Schroff R, Schanker HM, Weisman JD, Fan PT, Wolf RA (1981). Pneumocystis carinii Pneumonia and mucosal candidiasis in previously healthy homosexual men: evidence of a new acquired cellular immunodeficiency. N Engl J Med.

[37] Shiboski C, Hodgson T, Challacombe SJ (2011). Overview and research agenda arising from the Sixth World workshop on oral health and Disease in AIDS. Adv Dent Res.

[38] Shu W, Li C, Du F, Bai J, Duan K. A real-world, cross sectional study of oral lesions and their association with CD4 cell counts and HIV viral load in Yunnan, China. Medicine. 2020;99(40). 10.1097/MD.0000000000022416 PMC7535679 33019418

