# Supplementary figures and images for: Immune indices and oral health in patients infected with the human immunodeficiency virus
Source: BMC Oral Health. 2023 Dec 15;23:1009. doi: 10.1186/s12903-023-03752-y (PMC10724968; doi:10.1186/s12903-023-03752-y)

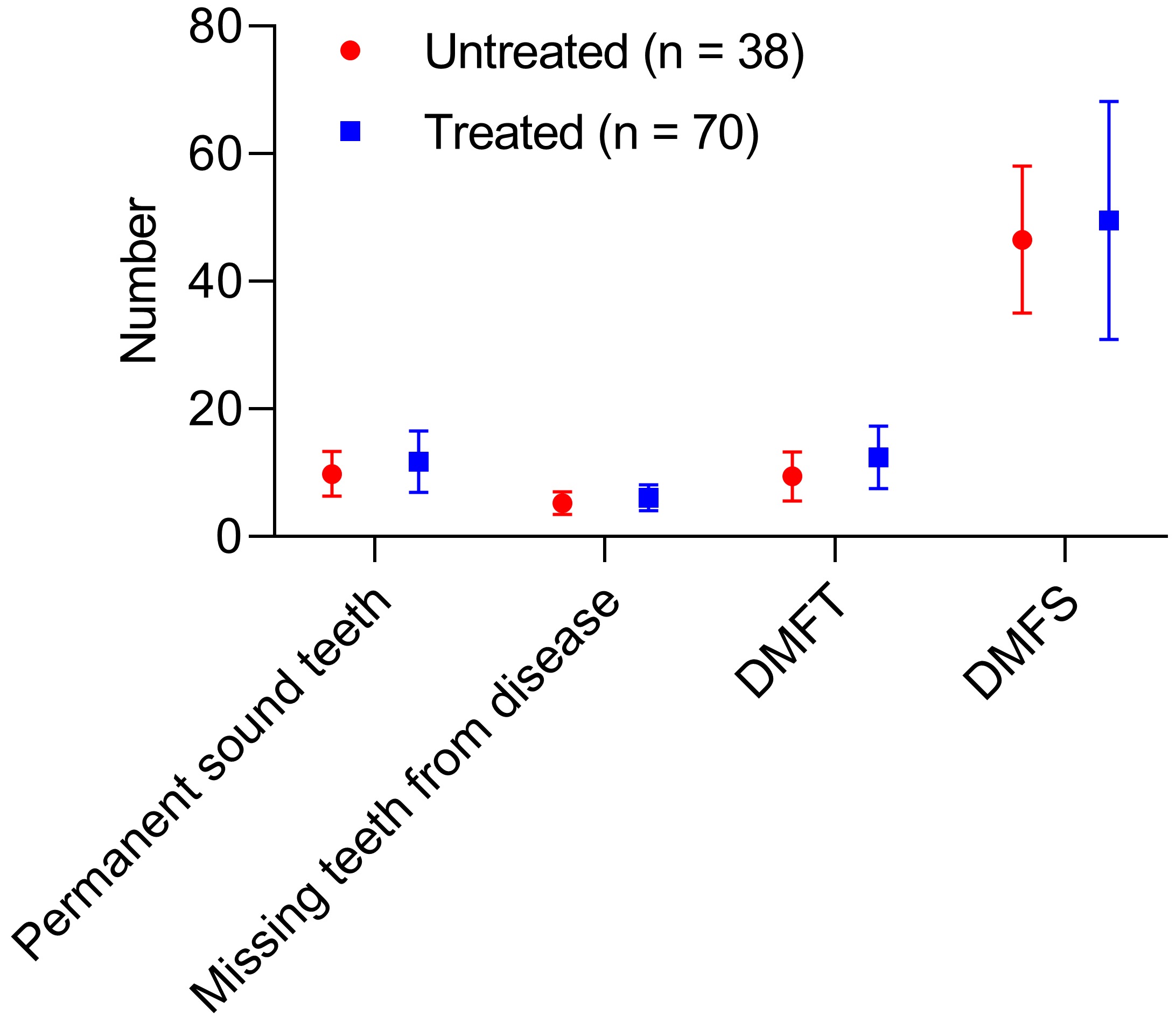

Supplement: Supplementary file 1 — Supplementary Material 1 [file 12903_2023_3752_MOESM1_ESM.tif]

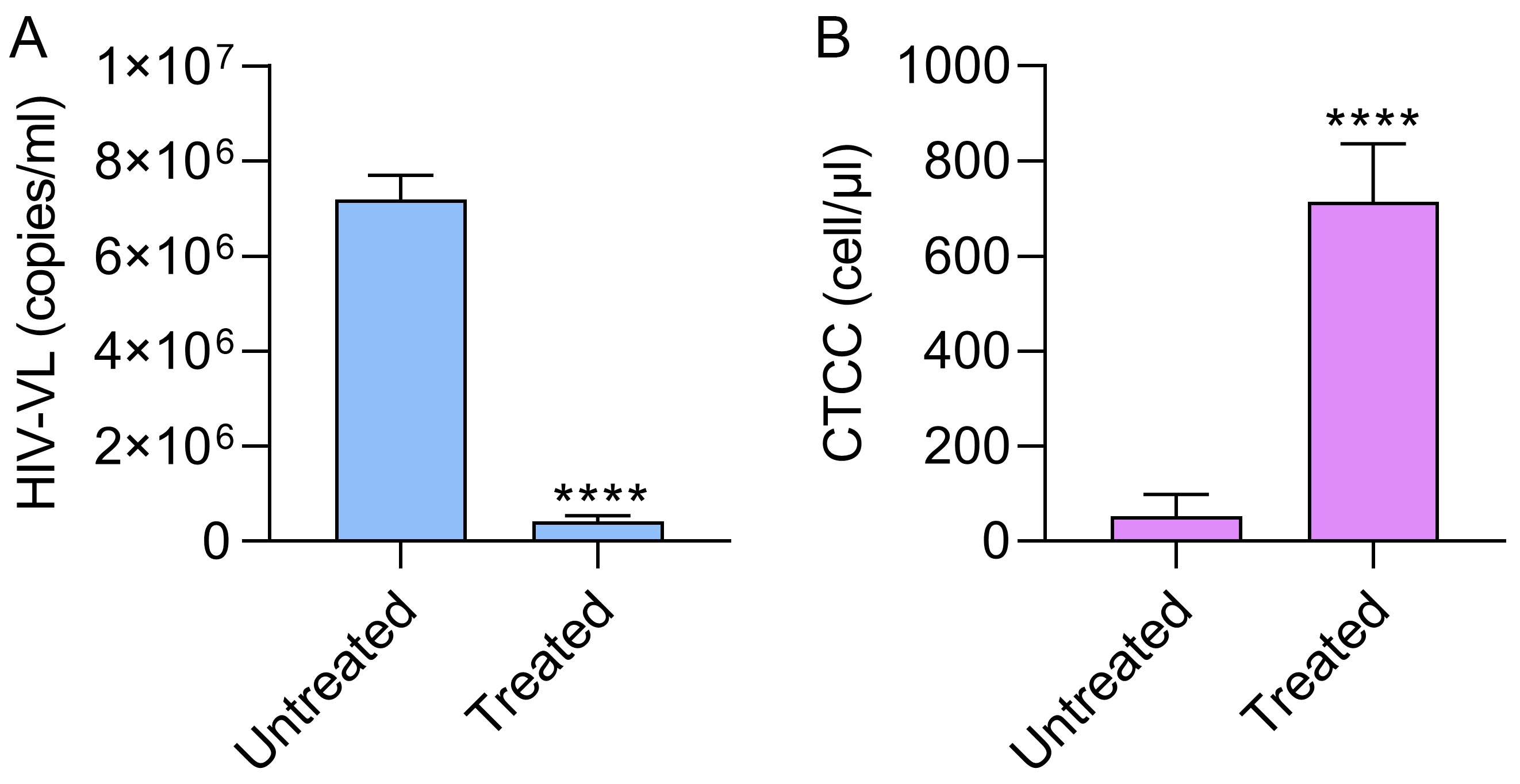

Supplement: Supplementary file 2 — Supplementary Material 2 [file 12903_2023_3752_MOESM2_ESM.tif]
