# Supplementary material for: Immune indices and oral health in patients infected with the human immunodeficiency virus
Source: BMC Oral Health. 2023 Dec 15;23:1009. doi: 10.1186/s12903-023-03752-y (PMC10724968; doi:10.1186/s12903-023-03752-y)
Supplement: Supplementary file 3 — Supplementary Material 3 [file 12903_2023_3752_MOESM3_ESM.docx]

**Figure S1**. Clinical index of dental health in treatment naive and experienced HIV infection patients. The number of permanent sound teeth, missing teeth from disease, DMFT, and DMFS was counted in treatment naive (untreated) and experienced HIV infection patients (treated).

**Figure S2**. HIV-VL and CTCC in treatment naive and experienced HIV infection patients. (A) HIV-VL (copies/ml) was counted in untreated and treated groups. (B) CTCC (cells/μl) was counted in untreated and treated groups.
